# CD19/CD20 dual-targeted chimeric antigen receptor-engineered natural killer cells exhibit improved cytotoxicity against acute lymphoblastic leukemia

**DOI:** 10.1186/s12967-024-04990-6

**Published:** 2024-03-13

**Authors:** Na Yang, Caili Zhang, Yingchun Zhang, Yuting Fan, Jing Zhang, Xiaojin Lin, Ting Guo, Yangzuo Gu, Jieheng Wu, Jianmei Gao, Xing Zhao, Zhixu He

**Affiliations:** 1 Tissue Engineering and Stem Cell Experiment Center, Guizhou Medical University (GMU), Guiyang, Guizhou China; 2 Department of Immunology, College of Basic Medical Sciences, Guizhou Medical University, Guiyang, Guizhou China; 3 Department of Biology, School of Basic Medical Sciences, Guizhou Medical University, Guiyang, Guizhou China; 4 Department of Gynecology, the Affiliated Hospital of Guizhou Medical University, Guiyang, China; 5 State Key Laboratory of Biotherapy and Cancer Center, Sichuan University, Chengdu, China; 6 School of Pharmacy, Zunyi Medical University, Zunyi, China; 7 Key Laboratory of Adult Stem Cell Translational Research, Chinese Academy of Medical Sciences), Guiyang, China; 8 Department of Pediatrics, the Affiliated Hospital of Zunyi Medical University, Zunyi, China

**Keywords:** Chimeric antigen receptor, Natural killer cells, Dual targets, mRNA, Acute lymphoblastic leukemia

## Abstract

**Background:**

Chimeric antigen receptor natural killer (CAR-NK) cells represent a promising advancement in CAR cell therapy, addressing limitations observed in CAR-T cell therapy. However, our prior study revealed challenges in CAR-NK cells targeting CD19 antigens, as they failed to eliminate CD19^+^ Raji cells in NSG tumor-bearing mice, noting down-regulation or loss of CD19 antigen expression in some Raji cells. In response, this study aims to enhance CD19 CAR-NK cell efficacy and mitigate the risk of tumor recurrence due to target antigen escape by developing CD19 and CD20 (CD19/CD20) dual-targeted CAR-NK cells.

**Methods:**

Initially, mRNA encoding anti-CD19 CARs (FMC63 scFv-CD8α-4-1BB-CD3ζ) and anti-CD20 CARs (LEU16 scFv-CD8α-4-1BB-CD3ζ) was constructed via in vitro transcription. Subsequently, CD19/CD20 dual-targeted CAR-NK cells were generated through simultaneous electrotransfection of CD19/CD20 CAR mRNA into umbilical cord blood-derived NK cells (UCB-NK).

**Results:**

Following co-electroporation, the percentage of dual-CAR expression on NK cells was 86.4% ± 1.83%, as determined by flow cytometry. CAR expression was detectable at 8 h post-electric transfer, peaked at 24 h, and remained detectable at 96 h. CD19/CD20 dual-targeted CAR-NK cells exhibited increased specific cytotoxicity against acute lymphoblastic leukemia (ALL) cell lines (BALL-1: CD19^+^CD20^+^, REH: CD19^+^CD20^−^, Jurkat: CD19^−^CD20^−^) compared to UCB-NK, CD19 CAR-NK, and CD20 CAR-NK cells. Moreover, CD19/CD20 dual-targeted CAR-NK cells released elevated levels of perforin, IFN-γ, and IL-15. Multiple activation markers such as CD69 and cytotoxic substances were highly expressed.

**Conclusions:**

The creation of CD19/CD20 dual-targeted CAR-NK cells addressed the risk of tumor escape due to antigen heterogeneity in ALL, offering efficient and safe 'off-the-shelf' cell products. These cells demonstrate efficacy in targeting CD20 and/or CD19 antigens in ALL, laying an experimental foundation for their application in ALL treatment.

**Supplementary Information:**

The online version contains supplementary material available at 10.1186/s12967-024-04990-6.

## Background

Acute Lymphoblastic Leukemia (ALL) is a hematological tumor arising from lymphoid progenitor cells of B-lineage or T-lineage [1], which due to abnormal growth of CD19^+^ precursor B cells and subsequent inhibition of normal hematopoiesis, resulting in anemia, thrombocytopenia, and neutropenia [2]. The peak incidence of ALL is between ages 2–5 in children [3], with 10% to 15% experiencing relapse despite achieving remission after first-line chemotherapy [4–6]. Refractory and relapse ALL (R/R-ALL) patients face a dismal prognosis with a 5-year overall survival rate below 20% [6]. Consequently, there is a need to develop novel, more efficient, and safer strategies to enhance the prognosis of R/R- ALL.

Chimeric antigen receptor T-cell (CAR-T) therapy has demonstrated efficacy in treating R/R-ALL by targeting CD19 [7], an ideal therapeutic target due to its high expression in ALL [8]. However, disease recurrence persists in a significant proportion (approximately 40 ~ 60%) of patients post CD19 CAR-T therapy [9]. Additionally, the labor-intensive and complex process of preparing CAR-T cells from a patient's own T cells may not be suitable for those with advanced tumors [10]. Allogeneic T cells pose the risk of complications such as Graft Versus Host Disease (GVHD) [11]. Furthermore, CAR-T cell therapy is associated with complications like cytokine release syndrome (CRS) and immune effector cell-associated neurotoxicity syndrome (ICANS) [12]. Exploring safe and effective treatment methods to prevent adverse reactions is thus essential.

CAR-NK therapy stands out as a promising alternative to CAR-T cells due to its distinct advantages [13]. In contrast to CAR-T cells, CAR-NK cells boast numerous benefits. NK cells are accessible through various methods, and they present a reduced risk of GVHD when employed in allogeneic settings due to the absence of major histocompatibility complex (MHC) restrictions [14, 15] and allogeneic NK cells may also be less prone to rejection by recipient alloreactive T cells [16]. This advantageous profile facilitates the development of safe 'off-the-shelf' cell products for CAR cell therapy. Furthermore, compared to activated CAR-T cells, which secrete IL-1α, IL-1Rα, IL-2, IL-2Rα, IL-6, TNF-α, MCP-1, IL-8, IL-10, and IL-15, which are highly correlated with CRS and ICANS, activated NK cells have a better safe profile, and their secretion of IFN-γ and GM-CSF relatively less likely contribute to the adverse reactions like CRS and ICANS [16–18].

Noteworthy is the initial clinical application of CD19 CAR-NK cells, demonstrating a compelling remission rate and safety in patients with R/R lymphoma (NCT03056339) [16]. Consequently, CAR-NK cell therapy emerges as a potential solution to multiple challenges faced by CAR-T cell therapy, positioning itself as a viable method in tumor immunotherapy.

In our previous research, we developed CD19 CAR-NK cells for the treatment of CD19^+^ tumors. However, in xenograft model mice, the administration of therapeutic cells and treatment cycles failed to eliminate CD19^+^ Raji tumors, indicating the potential for tumor-negative recurrence post-treatment. The efficacy of CD19 CAR-NK cell therapy may be compromised due to the downregulation or loss of CD19 antigen on tumor cell surfaces. Preliminary clinical trials with CD20 targeted therapy, such as Rituximab, have proven effective in cases of negative recurrence of CD19 antigen (NCT05618925). However, the heterogeneous expression of CD20 antigen on ALL cells poses a substantial challenge despite the proven efficacy of targeted drug therapy in treating ALL [19].

Given the downregulation and heterogeneity of tumor antigen expression, simultaneously targeting CD19/CD20 antigens on ALL cell surfaces using CAR-NK cells could represent an effective strategy. This approach has the potential to enhance CAR-NK cell targeting, diminish negative tumor recurrence, and mitigate the risk associated with single antigen heterogeneity.

**Fig. 1:**
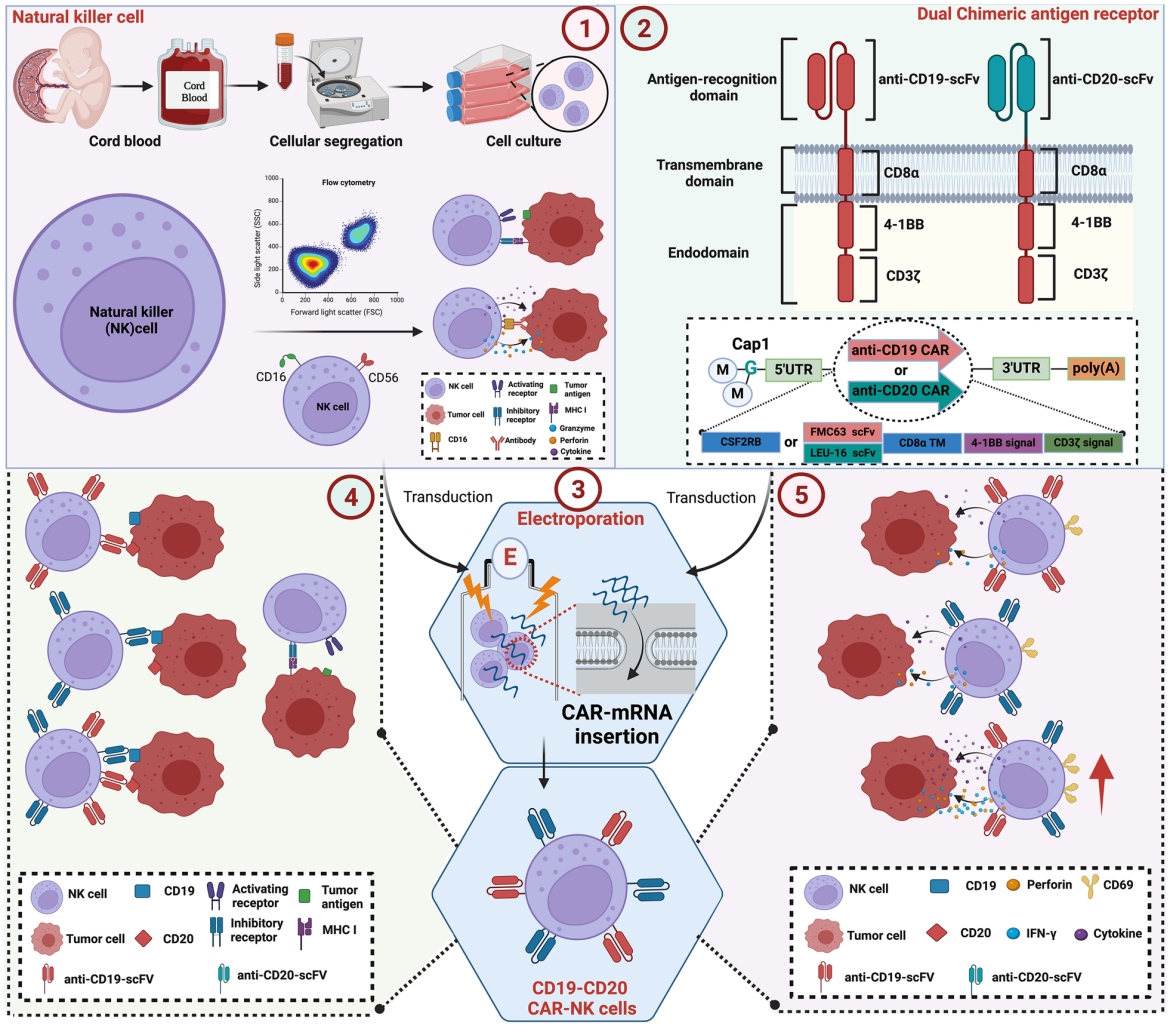
Preparation and mechanism of CD19/CD20 dual-targeted CAR-NK cell. ①: Ex-vivo expanded natural killer cells are derived from umbilical cord blood. ②: The mRNA encoding anti-CD19 CARs (FMC63 scFv-CD8α-4-1BB-CD3ζ) and anti-CD20 CARs (LEU16 scFv-CD8α-4-1BB-CD3ζ) were constructed by IVT. ③-④: CD19/CD20 dual-targeted CAR-NK cells were generated by simultaneous electroporation of CAR-mRNA into UCB-NK cells derived from umbilical cord blood, which specifically recognizes CD19^+^ and/or CD20^+^ ALL cells. ⑤: Once activated, CAR-NK binds to the target antigen and then lyses tumor cells by releasing perforin and IFN-γ. *CAR* chimeric antigen receptor, *IVT *in vitro transcription, *UCB* umbilical cord blood, *NK* natural killer cells

Building upon this background, our study devised two CAR molecules targeting CD19 and CD20 antigens on ALL cells, subsequently optimizing and refining their design. By employing in vitro transcription (IVT) [20], mRNA encoding anti-CD19 CARs (FMC63 scFv-CD8α-4-1BB-CD3ζ) and anti-CD20 CARs (LEU16 scFv-CD8α-4-1BB-CD3ζ) were produced (Fig. 1). CD19/CD20 dual-targeted CAR-NK cells were generated through the simultaneous electroporation of CAR mRNA into cord blood-derived NK cells (UCB-NK). Our study's findings demonstrate the feasibility of electro-transferring multiple mRNAs encoding CARs simultaneously, resulting in a substantial number of high-expression dual-target CAR-NK cells. These cells proved effective in recognizing and specifically clearing BALL-1 (CD19^+^CD20^+^) and REH (CD19^+^CD20^−^) ALL cells, even at low effector-target ratios. Moreover, CD19/CD20 dual-targeted CAR-NK cells exhibited high expression of the cell activity marker CD69 and secreted significant amounts of cytotoxic substances.

This study marks the first efficient preparation of CD19/CD20 dual-targeted CAR-NK cells derived from cord blood. These cells demonstrated effectiveness in eliminating double-positive (CD19^+^CD20^+^) and single-positive (CD19^+^ and/or CD20^+^) tumor cells, presenting a novel advancement for the clinical application of dual-target CAR-NK cell therapy in ALL treatment.

## Materials and methods

### Cell lines and cell culture conditions

Unless otherwise specified, all cell lines and reagents were procured from the American Tissue Culture Collection (ATCC, Manassas, VA, USA). Human acute lymphoblastic leukemia cell lines (BALL-1, REH, SUP; B15, NALM-6, CCRF, and Jurkat) as well as the human Burkitt lymphoma cell lines Raji and Daudi were cultured in RPMI-1640 medium (Gibco Invitrogen, USA). The culture medium was supplemented with 10% heat-inactivated fetal bovine serum (FBS; Biological Industries, IL) and 1% Penicillin–Streptomycin Solution (Gibco Invitrogen). Cultures were maintained in a humidified atmosphere at 37 °C with 5% CO_2_.

One million tumor cells were harvested from the culture, washed twice in cold Phosphate Buffer Saline (PBS; Gibco Invitrogen), and then pelleted at 1000 rpm for 5 min. Surface expression of CD19 and CD20 antigens on different tumor cells was detected by staining with human block Fc-receptors (2.5 μL, BioLegend, San Diego, USA) for 20 min at 4 °C, followed by a single wash and subsequent staining with anti-CD19-PE (5 μL, BioLegend, clone: H1819) or anti-CD20-APC antibodies (5 μL, BioLegend, clone:2H7) for 30 min at 4 °C. Non-staining tumor cells served as negative controls. After two washes, cells were resuspended in 200 μL PBS for quantitative analysis by flow cytometry.

### Ex vivo expansion of UCB-NK cells

All studies involving UCB-NK cells received approval from the Ethical Committee of Guizhou Medical University, Guizhou, China (2022–82). Umbilical cord blood mononuclear cells (UCB-MNCs) from healthy donors underwent density gradient centrifugation using Ficoll-Paque Plus (TBD, Tianjin, CHN). Subsequently, these cells were co-cultured with γ-irradiated K562 cell line expressing membrane-bound IL-21 (K562-mbIL-21) in a serum-free medium (ZhongYing Biology, Hangzhou, CHN). The medium was supplemented with 5% FBS and 200 IU/mL rhIL-2 (recombinant human interleukin-2; Kingsley Pharmaceutical Corporation). After 14 days of stimulation, cells were harvested for subsequent experiments.

For the analysis of UCB-NK cell phenotype and function in the co-culture system, one million cells were collected, washed in cold PBS, and then pelleted at 350 g for 5 min. Anti-CD3-FITC (5 μL, eBioscience, clone: OKT3), anti-CD16-APC (5 μL, clone: 3G8), and anti-CD56-PE (5 μL, clone: HCD56) antibodies were used to determine the purity of the NK cell population according to manufacturers’ instructions. The expression of the activity marker CD69 was determined using anti-CD69-FITC (5 μL, clone: FN50) antibody (all cell staining reagents for flow cytometry were from BioLegend). After two washes, all cells were resuspended in 200 μL PBS for quantitative analysis by flow cytometry. Each in vitro experiment was independently repeated with UCB-NK cells from different donors; UCB-NK cells were never pooled.

### Construction of CARs-expression plasmid, in vitro transcription of mRNA

To construct second-generation anti-CD19 CAR and anti-CD20 CAR, we designed the DNA fragment through in silico assembly (Fig. 3A). This fragment consisted of the optimized FMC63 scFv or LEU16 scFv sequence conjugated with CD8α hinge and transmembrane domain, 4-1BB cytoplasmic domain, and CD3ζ cytoplasmic domain. The optimized constructs of FMC63 scFv or LEU16 scFv sequence were minimally different from the initial constructs, except for the flexible linker connecting the variable heavy and light chains of the scFv binding domain, employing the (G_4_S)_3_ linker in FMC63 and LEU16. The CAR sequence commenced with a signal peptide (SP)- CSF2RB (Fig. 3B).

The CAR sequences described above were integrated into a synthetic vector, pVSIVT, situated between XhoI and NotI sites. The pVSIVT expression vector underwent modification, incorporating a T7 promoter, an alpha-globin 3' UTR sequence, and a 120-nt poly (A) sequence. The vector was linearized utilizing BspQI (Hongene, Shanghai, CHN) at the poly(A) terminus and utilized as the template for in vitro transcription of RNA conducted by T7 RNA polymerase (Hzymes Biotech, Wuhan, CHN). Notably, me1Ψ-UTP (Glycogene, Wuhan, CHN) was utilized as a substrate in lieu of UTP. Capping was achieved using vaccinia capping enzyme and 2'-O-methyltransferase (Hzymes Biotech, Wuhan, CHN). Subsequently, mRNA purification occurred employing oligo (dT)30 magnetic beads (Vdobiotech, Suzhou, CHN), and its quality was assessed through denaturing agarose gel electrophoresis. The RNA pellets were re-suspended in RNase-free water and stored at −80 °C.

### Generation of CAR-NK cells through mRNA electroporation

To generate CD19 CAR-NK, CD20 CAR-NK and CD19-CD20 CAR-NK cells, per 10^6^ expanded UCB-NK cells were combined with 3 μg of anti- CD19 CAR-mRNA and/or 3 μg anti-CD20 CAR-mRNA. These were then (co-)electroporated using the *Celetrix Electroporation* system (Celetrix Corporation, Manassas, Virginia, USA) under optimized parameters: volume: 120 μL, voltage: 640 V, pulse length: 30 ms, pulse once. Subsequently, NK cells were allowed to rest for a minimum of 4 h in a humidified atmosphere at 37 °C with 5% CO_2_ before downstream experiments.

To assess the cell surface expression of CAR, electroporated cells were stained with Biotinylated Human CD20 Full-Length protein (stock 50 μg/mL, 1:25 dilution, ACRO Biosystems, Beijing, CHN) for 60 min at 4 °C. This was followed by two washes in FACS buffer (PBA solution with 2% bovine serum albumin) and subsequent staining with allophycocyanin (APC)-conjugated Streptavidin Protein (stock: 200 μg/mL, 1:200 dilution, ACRO Biosystems) and FITC-Labeled Monoclonal Anti-FMC63 Antibody (stock: 50 μL, 1:50 dilution, ACRO Biosystems, clone: Y45) (or PE-Labeled Monoclonal Anti-FMC63 Antibody (stock: 50 μL, 1:50 dilution, ACRO Biosystems, clone: Y45)) for 60 min at 4 °C. Non-transduced cells and EGFP-mRNA transduced cells were stained with Streptavidin Protein only for use as negative controls. All stained cells were washed and resuspended in 200 μL PBS, subjected to flow cytometry acquisition, and analyzed using Flowjo10.

### Cytotoxicity analysis of CAR-NK cells

Unless otherwise noted, all analyses of CAR-NK cells are conducted after they have rested for 8 h post-electroporation. For the cytotoxicity analysis of CAR-NK cells, the Cell Counting Kit-8 reagent (CCK-8; Dojindo, JAP) was employed to examine the cytolytic activity against ALL cell lines. Target cells and CAR-modified NK cells (5 × 10^4^ cells each)were distributed in a U-bottom 96-well plate (100 μL volume) at different E: T ratios (E: T = 1:1/2:1/5:1) and incubated for 4 h at 37 °C with 5% CO_2_. CCK-8 reagent was then added to each well, and the optical density at 450 nm wavelength was analyzed on a *Synergy H1 Microplate Reader* (BioTek, Vermont, USA). Control groups were established to measure the effector group (only effector cells added), target group (only target cells added), and medium group (no cells added). The proportion of cytotoxicity was calculated based on the fraction of live cells, and the percent specific lysis (as Killing Rate (%)) was determined using the following formula:

$$ {\text{Killing}}\,{\text{Rate}}\,\left( \% \right)\, = \,\left( {{1}\, - \,\frac{{{\text{Experimental group }}\left( {{\text{OD}}} \right) - {\text{Effector group }}\left( {{\text{OD}}} \right) - {\text{Medium group }}\left( {{\text{OD}}} \right)}}{{{\text{Target group }}\left( {{\text{OD}}} \right) - {\text{ Medium group }}\left( {{\text{OD}}} \right)}}} \right)\, \times \,100 $$

### Flow cytometry

We employed the flow cytometer to determine the specific lysis of CAR-NK cells. In brief, we used CFSE (5,6-carboxyfluorescein diacetate, succinimidyl ester; Thermo Fisher Scientific, Waltham, MA) to trace tumor cells. The target cells were incubated in a complete medium with 5 mM CFSE at a final concentration of 10^6^ cells/mL for 20 min at 37 °C and washed twice with the medium. Subsequently, the target cells were re-suspended at a final concentration of 1 × 10^6^ cells/500 μL of medium. Different CAR-NK cells were serially diluted to achieve E:T ratios of 1:1 and co-cultured with the target cells for 4 h at 37 °C with 5% CO_2_. Following this procedure, cells were collected and stained using PI dye (Propidium Iodide; Thermo Fisher Scientific) before flow cytometry.

### Functional analysis of CAR-NK cells

Human perforin, IFN-γ, and rhIL-15 secretion of CAR-NK cells were determined by enzyme-linked immunosorbent assay (ELISA). BALL-1 cells and NK cells or CAR-NK cells were distributed in a 96-well plate with a volume of 100μL at E: T ratios = 1:1 and incubated for 4 h at 37 °C with 5% CO_2_. All samples were in triplicate. Subsequently, co-cultured supernatant was collected, and the levels of perforin, IFN-γ, and IL-15 were detected according to the protocols of ELISA kits (Enzyme-linked Biotechnology, Shanghai, CHN/ MULTISCIENCES, Zhejiang, CHN). Unless otherwise noted, all data presented represent three or more independent experiments.

For flow cytometry analysis of IFN-γ and CD107a expression, CAR-NK/NK cells were cocultured with target cells (BALL-1 cells) at E: T = 1:1, using BFA (Brefeldin A; MedChemExpress, NJ, USA) for 4 h at 37 °C with 5% CO_2_. After incubation, cells were stained with anti-IFN-γ-APC antibody (5 μL, eBioscience, clone: B27) or anti-CD107a-FITC antibody (5 μL, eBioscience, clone: eBioH4A3) and used the Cytofx/Cytoperm Kit (BD Biosciences) for 30 min at 4 °C. All stained cells were washed and resuspended in 200 μL PBS, subjected to flow cytometry acquisition, and analyzed using Flowjo10.

### Statistical analyses

We performed statistical analyses using GraphPad Prism version 9 software. The statistical significance of in vitro results was analyzed using a two-tailed, unpaired, homoscedastic Student *t*-test. Data are presented as means ± SD unless otherwise noted. All *p* < 0.05 were considered statistically significant.

## Results

### Ex vivo-expanded natural killer cells derived from umbilical cord blood

NK cells have demonstrated potential in adoptive cell therapy due to their anti-tumor activity without prior sensitization [15]. ‘Off-the-Shelf’ products are essential to enhance the adoptive NK cell therapy applications. Researchers have revealed that umbilical cord blood (UCB) contains a higher percentage of NK cells, approximately 15 ~ 30% [21, 22].

**Fig. 2:**
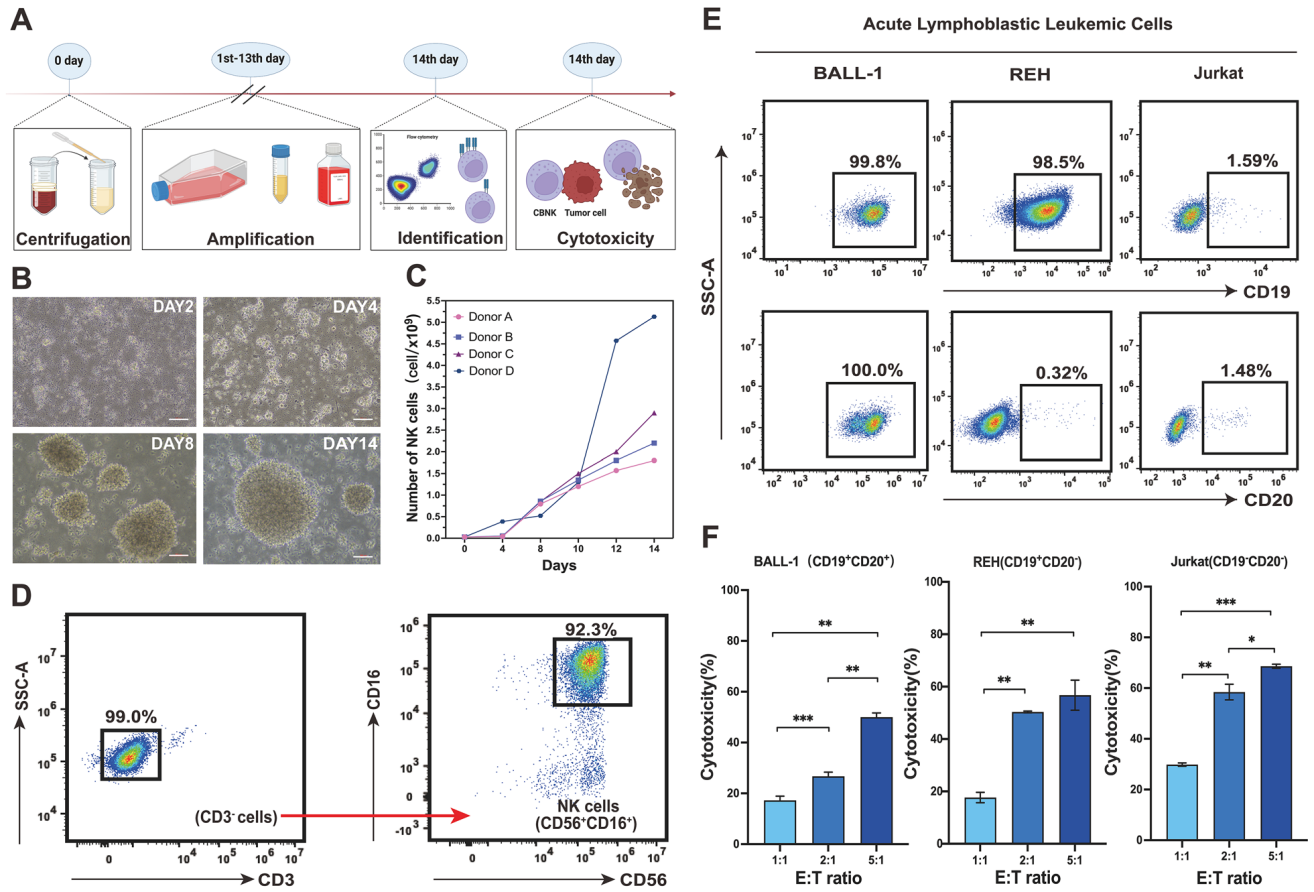
Ex vivo expansion and identification of UCB-NK*.* **A** Schematic diagram of ex vivo-expanded UCB-NK cells. **B** Morphology of NK cells at different cultured times observed via an optical microscope (Scale Bar = 200 μm). **C** Growth curves of UCB-NK cells from different donors. **D** Purity of UCB-NK cells as determined by flow cytometry. **E** Expression of CD19 and CD20 antigens on the surface of different acute leukemia cells. **F** Cytotoxic effects of UCB-NK cells on different acute lymphoblastic leukemia cells. Data are presented as mean values and S.D. of triplicate samples (*p < 0.05, **p < 0.01, ***p < 0.001, n = 3). UCB-NK, NK cells derived from umbilical cord blood

Therefore, in this study, we expanded UCB-NK cells via a feeder-cell system to obtain numerous high-purity and cytotoxicity NK cells. The results showed that UCB-MCN were obtained from healthy donors by density gradient centrifugation, followed by co-culturing with K562-mbIL-21 cells in a medium containing 200 IU/mL IL-2 to amplify UCB-NK cells (Fig. 2A). Compared with the initial cells, the UCB-NK cells became more full and round, while cell colonies appeared (Fig. 2B). A substantial amount of NK cells were successfully obtained by feeder-cell method (Fig. 2C). Notably, the cells obtained from donor group D exhibited a higher absolute count compared to the other three groups possibly due to the higher proportion of NK cells present in the culture system (Fig. 2C). Next, the purity of UCB-NK cells after culturing was assessed via flow cytometry (Additional file 1: Fig. S1a). The cultured system demonstrated a predominant presence of CD3^−^CD16^+^CD56^+^ NK cells, exceeding 90% (Fig. 2D). Concurrently, the expression of CD69, an early activation marker for NK cells, exhibited a significant increase at various time points throughout the culture cycle. This increase positively correlated with the activation of NK cells (Additional file 1: Fig. S1d). Co-cultivation with target cells such as BALL-1 (CD19^+^ CD20^+^), REH (CD19^+^ CD20^−^), and Jurkat (CD19^−^ CD20^−^) or other blood tumor cells (Fig. 2E and Additional file 1: Fig. S1b) at diverse effector-target (E: T) ratios for 4 h revealed the cytotoxicity of UCB-NK cells against various blood tumor cells. Notably, the cytotoxicity of UCB-NK cells increased proportionally with the elevation of the E:T ratio (Fig. 2F and Additional file 1: Fig. S1c), establishing a positive correlation between the killing rate and the E:T ratio.

However, the cytotoxic effect of UCB-NK cells was less conspicuous at low E:T ratios (E: T = 1:1), suggesting a nuanced relationship between cytotoxicity and the precision of NK cell identification and specificity (Fig. 2F and Additional file 1: Fig. S1c). Moreover, the moderate cytotoxic effect at low E:T ratios prompts consideration of the role of accurate identification and specificity of NK cells. Genetic engineering of NK cells with CARs empowers NK cells to selectively recognize and lyse tumor cells. This is achieved by releasing killing mediators and inducing apoptosis in the target cells, thereby enhancing the specificity and efficacy of adoptive NK therapy.

### Construction of second-generation CARs targeting CD19/CD20 antigens

**Fig. 3:**
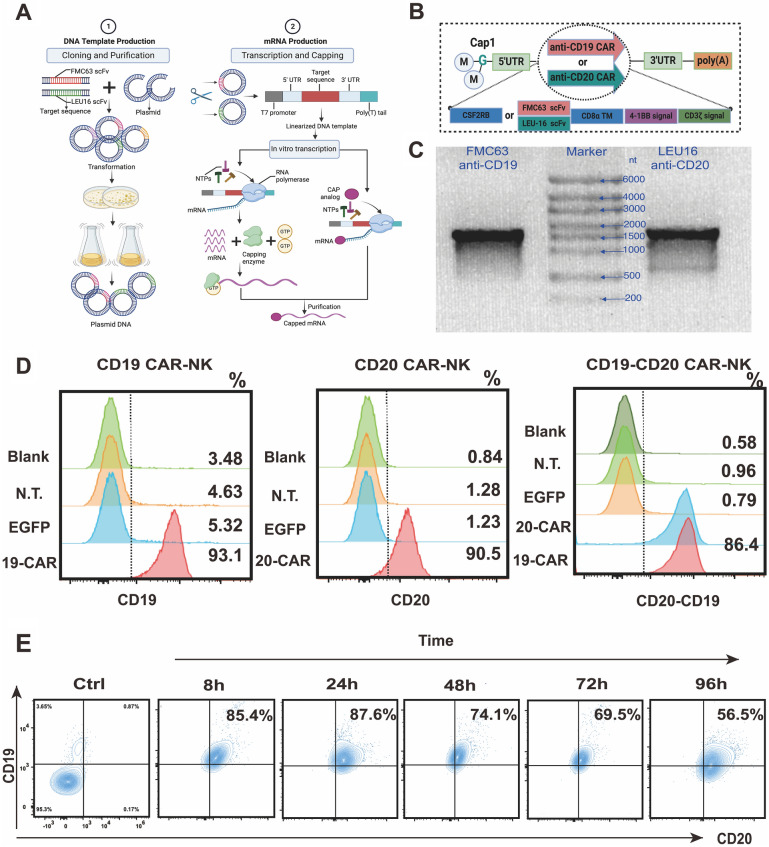
Construction and identification of the second-generation CARs targeting CD19 and CD20 antigen. **A** Schematic illustration of the construction of mRNA encoding CD19 CARs and CD20 CARs utilized IVT. **B** Schematic diagrams of the CD19 CAR-mRNA and the CD20CAR-mRNA. FMC63(anti-CD19) scFv and LEU16 (anti-CD20) scFv were linked to the CD8α hinge and transmembrane domain, the 4-1BB (CD137) signaling domain, and the CD3 zeta signaling domain, respectively. **C** Denatured agarose gel electrophoresis of the CAR-mRNA. Lane (1) represents the CD19 CAR-mRNA group, lane (3) represents the CD20 CAR-mRNA group, and lane (2) represents mRNA Marker (6000nt). **D** Detection of CAR expression on NK cells by flow cytometry. CAR expression was detected by using one or two of four staining reagents: PE-Labeled monoclonal anti-FMC63 Antibody (column 1), Biotinylated Human CD20/MS4A1 full length protein followed by Streptavidin Protein-Alexa Fluor 647 (column 2), or FITC-Labeled Monoclonal Anti-FMC63 Antibody (column 3). The expression percentage of CAR on NK cells is noted on the right of each histogram. EGFP-transduced cells served as an additional negative control. **E** CD19 CARs and CD20 CARs expression kinetics on dual-target CAR-NK over 4 days. *IVT *in vitro transcription, *scFv* single-chain fragment variable regions, *Cap1* Cap1 structure, *UTR* untranslated region

CARs play a pivotal role in enhancing immune cell targeting and killing abilities by translating extracellular signals into intracellular responses [23]. The antigen-binding specificity of CARs relies on the single-chain variable fragment (scFv), comprising the variable light chain (VL) and variable heavy chain (VH) of monoclonal antibodies connected via a short linker [24]. In this investigation, we designed and generated second-generation anti-CD19-CARs and anti-CD20-CARs, incorporating signal peptide (SP), anti-CD19 (FMC63) scFv, and anti-CD20 (LEU16) scFv as the extracellular domains of CAR molecules. These were linked to the CD8α hinge and transmembrane domain, 4-1BB costimulatory endo-domain, and CD3ζ signaling domain (Fig. 3A, B).

Studies have demonstrated that incorporating flexible linkers of appropriate length enhances the stability of scFv and CAR structures, critical for the effectiveness and durability of CAR-NK cells [24]. Building upon this knowledge, we optimized the scFv of the CAR molecule used in this study, with the linker between VL and VH of scFv set to (G_4_S)_3_. This linker structure, based on glycine (Gly) and serine (Ser) repeat polypeptides ((Gly_4_Ser)_3_ or (Gly_4_Ser)_4_), aims to provide flexibility while minimizing interference with the spatial folding of attached protein domains [25], thereby enhancing CAR-NK cell function.

Here, we designed and constructed second-generation CAR molecules targeting CD19 and CD20 antigens (Fig. 3A). The mRNA encoding anti-CD19 CARs and anti-CD20 CARs (CD19 CAR-mRNA/CD20 CAR-mRNA) were obtained by IVT and validated through denaturing agarose gel electrophoresis (Fig. 3C). Subsequently, we modified UCB-NK cells with CD19 CAR-mRNA and CD20 CAR-mRNA through electroporation, generating CD19 CAR-NK cells, CD20 CAR-NK cells, and CD19/CD20 dual-targeted CAR-NK cells. As depicted in Additional file 1: Fig. S2, efficient CAR expression on NK cells was achieved by electroporating 1 μg CAR-mRNA with 10^6^ NK cells. Remarkably, CAR expression exhibited a negative correlation with mRNA electro-transfected concentration, decreasing with excessive loading of CAR-mRNA (Additional file 1: Fig. S2). Based on this observation, we explored and optimized the co-transfection concentration of CAR-mRNA to generate CD19-CD20 CAR-NK cells with high CAR expression. We successfully developed numerous CD19/CD20 dual-targeted CAR-NK cells by co-electro-transfecting anti-CD19-CAR mRNA (3 μg) and anti-CD20-CAR mRNA (3 μg) into NK cells (10^6^ cells), with more than 86% of NK cells expressing both CARs (Fig. 3D).

A prior study noted a gradual decline in CAR expression within 4 days after mRNA electroporation into NK cells, eventually returning to baseline levels [26]. Interestingly, as illustrated in Fig. 3E, the expression of anti-CD19 and anti-CD20 CARs peaked at 24 h and remained efficient for at least 96 h, ranging from 87 to 56%. This observed stability may be attributed to the optimized CAR structure, achieving a balance between effective transfection and sustained expression.

### Enhanced cytotoxicity of CD19/CD20 CAR-NK cells through specific recognition of CD19 and/or CD20 antigens

The results demonstrate the surface expression of CD19/CD20 antigens on various malignant hematological tumor cells, as confirmed by flow cytometry (Fig. 2E and Additional file 1: Fig. S1b). Three ALL cells, namely BALL-1 (CD19^+^CD20^+^), REH (CD19^+^CD20^−^), and Jurkat (CD19^−^CD20^−^), were selected for further experimentation based on their expression levels.

**Fig. 4:**
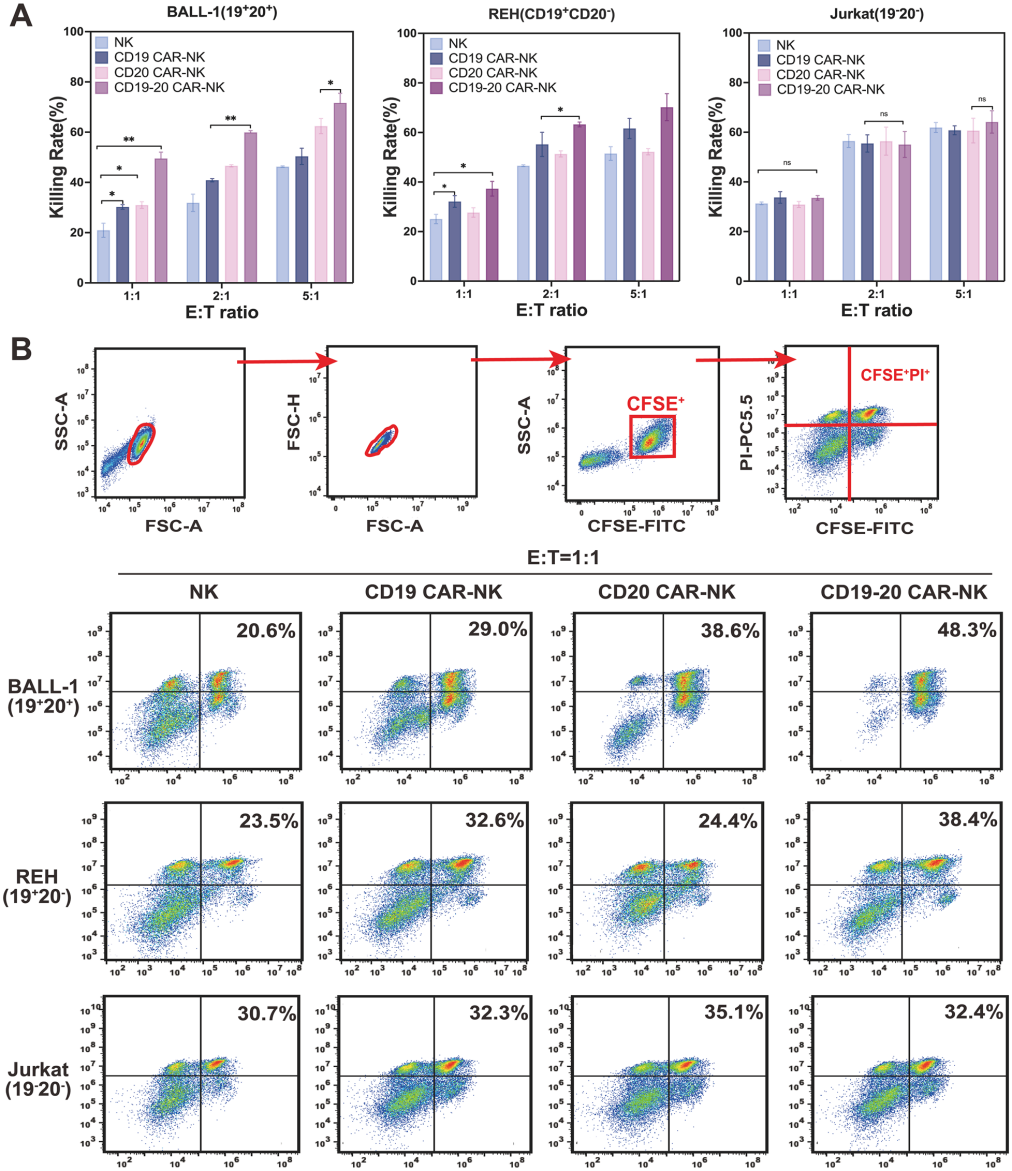
Specific cytotoxicity of CD19/CD20 dual-targeted CAR-NK cells against CD19 positive/CD20 positive acute lymphoma cells. **A** Detection of cytotoxicity of dual-target CAR-NK cells and single-target CAR-NK cells on acute lymphoma cells (BALL-1, REH, and Jurkat) at different E:T ratios. Data are presented as mean values and S.D. of triplicate samples (*p < 0.05, **p < 0.01, n = 3). **B** Death of BALL-1, REH, and Jurkat cell lines was detected by flow cytometry (CFSE/PI). Nucleated cell gates were gated according to the size and complexity of the samples (FCS-A and SSC-A, respectively). Nucleated cells were further gated in FSC-A and FSC-H to screen for single cells and exclude doublets. From the single cell gate, tumor cells were defined as CFSE^+^. The CFSE^+^PI^+^ population was defined as dead tumor cells from the CFSE^+^ gate

In comparison to different ALL target cells, CD19/CD20 CAR-NK cells exhibited notable and specific cytotoxicity against BALL-1 and REH cells, unlike conventional NK cells. Conversely, all CAR-NK cells displayed similar cytotoxicity to NK cells in the absence of CD19 or CD20 antigen stimulation (Jurkat cells) (Fig. 4A). Notably, despite previous results suggesting a potential competitive decrease in CAR expression with simultaneous electro-transfection of two CAR-mRNAs (Fig. 3D and Additional file 1: Fig. S2), CD19/CD20 CAR-NK cells demonstrated slightly more potent cytotoxicity against BALL-1 and REH cells than single-target CAR-NK cells at a lower E:T ratio (Fig. 4A).

Moreover, it was observed that CD19/CD20 CAR-NK cells exhibited effective lysis of REH (CD19^+^CD20^−^) cells, in contrast to the weak cytotoxicity of CD19 CAR-NK cells (Fig. 4A). This indicates that the dual-targeting CAR domain can recognize and bind tumor cells with just one homologous antigen, enabling CD19/CD20 CAR-NK cells to lyse CD19^+^CD20^−^ or CD19^−^CD20^+^ tumor cells in case of CD19 or CD20 tumor escape. Importantly, this enhanced cytotoxicity compared favorably to single antigen-expressing counterparts (CD19 CAR-NK and CD20 CAR-NK), confirming that dual-CAR loading is not detrimental to the cytotoxic function (Fig. 4A).

To quantitatively assess the antitumor effects of dual-target CAR-NK against leukemia, dead target tumor cells, identified as CFSE^+^PI^+^ cells, were analyzed by flow cytometry. CFSE-labeled tumor cells were added to E:T ratios of 1:1, and the cells were subsequently stained with PI (Propidium Iodide) (Fig. 4B). These findings indicate that CD19/CD20 dual-targeted CAR-NK cells, generated through the electroporation of CAR-mRNA into UCB-NK cells, can specifically recognize tumor cells expressing CD19 and/or CD20 antigens, leading to effective lysis.

### CD19/CD20 dual-targeted CAR-NK cells demonstrate heightened activation and increased cytokine secretion

**Fig. 5:**
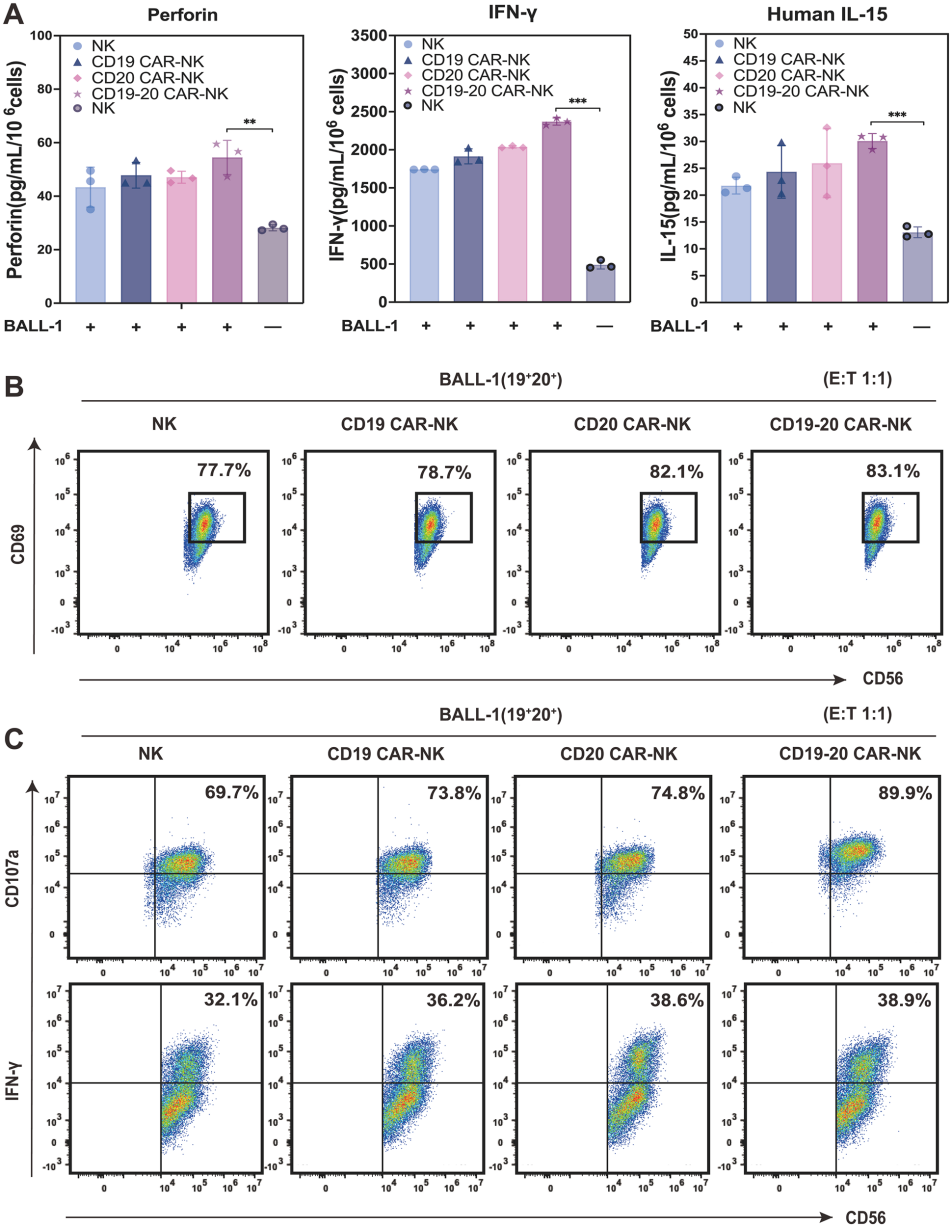
Antitumor activity of CAR-NK cells. **A** The levels of perforin, IFN-γ, and IL-15 secreted by NK and CAR-NK cells as detected by ELISA (1:1 E: T ratio; n = 3; **p < 0.01, ***p < 0.001). **B** Expression of CD69 on NK/CAR-NK cells as detected by flow cytometry during 4 h of co-culture at a 1:1 E: T ratio. **C** Intracellular expression of CD107a and IFN-γ on NK/CAR-NK cells as detected by flow cytometry during 4 h of co-culture at a 1:1 E: T ratio

To further investigate the antitumor efficacy of CAR-NK cells, we assessed the release of perforin, IFN-γ, and IL-15 from CAR-NK cells when stimulated by BALL-1 cells using ELISA. When co-cultured with BALL-1 cells, both NK cells and single-target CAR-NK cells exhibited elevated secretion levels of perforin, IFN-γ, and IL-15 in comparison to non-tumor-activated NK cells. Remarkably, CD19/CD20 dual-targeted CAR-NK cells displayed a significant augmentation in the secretion of these cytokines (Fig. 5A). Noteworthy is the observation that CD19/CD20 dual-targeted CAR-NK cells exhibited the highest cytokine secretion, even in the absence of activation (Additional file 1: Fig. S3a). Intriguingly, distinct CAR-NK cells displayed varying levels of cytokine secretion in a tumor-free environment (Additional file 1: Fig. S3a). This indicates that the dual-targeting CAR domain can recognize and bind tumor cells with just one homologous antigen, enabling CD19/CD20 CAR-NK cells to lyse CD19^+^CD20^−^ or CD19^−^CD20^+^ tumor cells in case of CD19 or CD20 tumor escape. We posit that the enhanced targeting facilitated by the dual-target CAR structure amplifies NK cells' recognition of target antigens, leading to intensified signal transduction and cellular activation.

Subsequently, we evaluated the expression of the activation marker CD69, the degranulation marker CD107a, and the intracellular cytokine IFN-γ throughflow cytometry. CD69, an early surface antigen expressed post-lymphocyte activation, serves as a pivotal marker for NK cell activation. Following the interaction of CAR-NK cells with tumor cells, the up-regulation of CD69 expression typically accompanies cell activation. As depicted in Fig. 5B, the expression of CD69 in CAR-NK cells increased compared to NK cells under specific tumor load conditions, with the highest activation level observed in CD19/CD20 dual-targeted CAR-NK cells. Interestingly, no significant difference in CD69 expression was noted in the absence of tumor cells (Additional file 1: Fig. S3b). The antitumor activity of NK cells was further delineated by the expression of CD107a and IFN-γ. CAR-NK cells exhibited higher expression levels of CD107a and IFN-γ in a tumor-loaded environment compared to NK cells, with CD19/CD20 dual-targeted CAR-NK cells displaying the highest expression levels (Fig. 5C). Notably, even in the absence of tumor cells, dual-target CAR-NK cells maintained elevated expression levels of CD107a and IFN-γ (Additional file 1: Fig. S3c), aligning with the anticipated results mentioned earlier. These findings underscore the significant enhancement in activation and degranulation marker expression, as well as the secretion of cytotoxic granules and cytokines by CD19/CD20 dual-targeted CAR-NK cells, highlighting their pivotal role in NK cell activation.

## Discussion

Despite notable strides in remission and survival rates for ALL, patients undergoing conventional treatment still encounter a challenging prognosis [6]. Existing research demonstrates the potential benefit of CD19 CAR-T cell therapy in treating advanced B-lymphoid malignancies [27]. However, among the nine currently available CAR-T cell products, only one (Kymriah) has gained approval in Europe and the United States for R/R ALL patients under the age of 25, with a price tag of $475,000 [28]. The clinical application of CAR-T therapy faces limitations, primarily attributed to the functional dysfunction of autologous T cells, particularly in pre-chemotherapy or immunosuppressive patients, and the risk of GVHD caused by allogeneic T cells [11]. Conversely, allogeneic NK cells pose a lower risk of GVHD, enabling the production of “off-the-shelf” CAR products, potentially reducing treatment costs. Furthermore, the cytokines secreted by NK cells post-treatment are less likely to induce CRS and ICANS, rendering it a safer alternative [13]. According to the latest data from *ClinicalTrials.gov*, 54 clinical trials of CAR-NK are underway, with approximately 33.3% focused on CD19 CAR-NK, highlighting the significant potential of CAR-NK cell therapy in overcoming ALL.

While substantial progress has been achieved in treating ALL with CD19 CAR-NK cells, tumor cells have been observed to induce tumor-negative recurrence by down-regulating CD19 antigen expression on their surface [29]. Drugs targeting CD20 antigens, such as rituximab, a monoclonal antibody commonly used to treat lymphoma, also show similar recurrence due to CD20 being internalized from the surface of B cells after rituximab treatment [30, 31].

The simultaneous loss of both CD19 and CD20 antigens is improbable, as they are generally expressed on ALL cells [32]. When employing single-target CAR-NK cell therapy, heterogeneous target antigens may lead to tumor escape. Therefore, targeting both CD19 and CD20 antigens may be a feasible strategy to overcome tumor escape. Currently, different approaches are being explored to construct multi-targeted CAR structures, allowing simultaneous targeting of multiple antigens and extending the function of CAR-NK cells to several phenotypic subgroups of a tumor [33].

The following forms of implementation for constructing CAR-NK cells include [34]: (1) Constructing two types of CAR-NK cells, each targeting a distinct antigen, administered either in combination or sequentially; (2) Introducing NK cells with bicistronic vectors encoding two separate antigens on the same cell to produce two different CARs in the same NK cell; (3) Arranging the VL and VH chains of scFv in a different order to form two different structures, either tandem or circular, and obtaining Tan-CAR by encoding a single CAR carrier.; or (4) Simultaneously editing NK cells using two different CAR structures (co-transduction) will result in three subpopulations of CAR-NK cells consisting of both dual-target and single-target CAR-NK cells.

Designing dual-target CAR molecules is challenging, often requiring different constructs to effectively assess the impact of both scFv domains on the phenotype and function of resulting dual-target CAR-NK cells [35]. The ideal construct ensures that CAR-NK cells can perform functions for both target antigens [36]. Notably, the position of the corresponding scFv in CAR must be carefully adjusted to bind effectively with homologous antigens on target cells [35]. Our investigation centered on the CD19 and CD20 antigens in ALL. CD19, a single-pass transmembrane immunoglobulin-like molecule, resides external to the cell membrane, while CD20, a multi-pass transmembrane molecule, is identified by membrane markers [37, 38]. Due to these structural distinctions, targeting CD20 necessitates an elongated extracellular CAR domain, whereas CD19 requires a shorter one. In our research, we co-transfected NK cells with two distinct CAR structures, a strategy facilitating clinical-level production and justified by the molecular configuration of CARs. Our findings indicated robust expression of CD19/CD20 CAR eight hours post simultaneous electroporation, persisting until 96 h. It's noteworthy that refining the scFv structure of the targeted antigen molecule into a singular CAR molecule resulted in Tan-CAR. Tan-CAR possesses a reduced DNA footprint by approximately 40% [36], enhancing the packaging and transduction efficiency of viral vectors [39]. This heightened transduction efficiency is pivotal for the effective clinical application of CAR cells. Considering that CAR cell products are typically infused into polyclonal populations, pre-sorting CAR^+^ cells prove unnecessary. We employed mRNA encoding anti-CD19 and anti-CD20 CARs to modify NK cells, achieving over 90% transduction efficiency within a relatively short timeframe.

Research has demonstrated that the interconnection of the VH and VL chains in scFvs influences the affinity and specificity of CAR molecules toward their target epitopes [40]. Hence, various linker molecules, predominantly based on Gly and Ser repeat peptides such as (Gly_4_Ser)_3_, consists of three repetitions Gly-Gly-Gly-Gly-Ser [41], have successfully designed scFvs. These residues provide the linker with flexibility to minimize interference with the folding of attached protein domains [42]. Moreover, the optimal linker length, usually 15–20 amino acids, prevents aggregation, as excessively short linkers can induce non-antigen-dependent CAR aggregation, leading to tonic signaling, CAR-T depletion, and incapacitation [43]. The CAR molecular structure employed in this study is of the second-generation type, with optimized linkers connecting FMC63 scFv (anti-CD19) and lEU16 scFv (anti-CD20) to the CD8α transmembrane domain, 4-1BB costimulatory domain, and CD3ζ signal transduction domain through the hinge region. Notably, CAR molecules featuring 4-1BB as the signal domain exhibited superior in vivo proliferation and killing capabilities compared to those with CD28 [42]. The inclusion of CD3ζ, a signaling component of T cells, enhances CAR-T cell signaling function. However, it is crucial to tailor the CD3ζ gene sequence in CAR-NK cells for different therapeutic goals, optimizing therapeutic effects [13]. In comparison to earlier CD3ζ-based CAR-NK cells, current research places higher importance on NK cell-specific signal adaptors, particularly DAP10, DAP12, and 2B4 [44]. DAP10 serves as a co-stimulatory element, enhancing CAR-NK cell activity, while DAP12 and 2B4 augment the killing effect by activating cytotoxicity and cytokine secretion of NK cells.

NK cells for therapeutic applications can be derived from various sources [45]. Presently, the majority of clinical trials involving NK cells focus on those sourced from UCB, peripheral blood (PB), human embryonic stem cells (hESCs), induced pluripotent stem cells (iPSCs), and established cell lines such as the lymphoma-derived NK cell line NK-92. NK cells constitute approximately 10% of all lymphocytes in peripheral blood, whereas they make up 30% of lymphocytes in UCB. UCB, in particular, emerges as a promising reservoir of cytotoxic NK cells [46]. The UCB repository offers distinct advantages for donor selection based on specific human leukocyte antigen (HLA) types and NK receptor spectra. Irrespective of the chosen cell source, a substantial infusion of NK cells is required for a single treatment, and conventional cytokine-based cell expansion methods are both expensive and time-consuming. Notably, prior studies have utilized modified K562 leukemia feeder cell lines expressing membrane-bound cytokines to stimulate in vitro expansion of NK cells [47]. In our investigation, K562-MBIL-21 cells served as feeder cells [48]. Our results demonstrate that this amplification method yields a harvest of up to 3 × 10^9^ cells, with a positive NK phenotype rate exceeding 90% (Fig. 2C, D).

While we posit that maintaining a stable expression of CARs in primary NK cells is crucial for sustained in vivo anti-tumor effects, retrovirus transfection of primary NK cells poses certain risks, including the potential for gene insertion mutations [49]. In contrast, non-viral methods, such as electroporation transfection of mRNA, present several advantages, including low immunogenicity, the capacity to deliver large gene segments (> 100 kb), high transduction efficiency, biosafety, and reduced manufacturing costs [50]. In our study, CAR mRNA was electroporated into UCB-NK cells to generate CD19-CD20 CAR-NK. While short-term expression of CD19/CD20 CAR (5–7 days) may suffice to elicit significant cytotoxic effects of dual-target CAR-NK cells on tumor cells in vitro, its long-term efficacy in controlling tumor growth may be insufficient. Addressing this concern may necessitate multiple CAR-NK cell infusions, and future investigations should focus on optimizing their sustained efficacy. Notably, the FDA recently approved the first CAR-T cell therapy targeting BCMA. However, increased BCMA expression during B-cell differentiation, especially in late memory B cells and normal plasma cells, heightens the risk of off-target effects (i.e., targeting normal cells instead of the tumor) [51]. Hence, CAR-modified NK cells with short-term expression appear more advantageous than long-term persistent expression, mitigating the risk of therapeutic off-target toxicity associated with prolonged dysplasia.

## Conclusion

Our study successfully generated CD19/CD20 dual-targeted CAR-NK cells for combating ALL, thereby reducing the risk of tumor escape resulting from tumor antigen heterogeneity, down-regulation, or loss of antigen expression in ALL. These dual-target CAR-NK cells represent efficient and safe 'off-the-shelf' cell products. Our findings indicate that dual-target CAR-NK cells can simultaneously target CD20 and/or CD19 antigens in ALL, exhibiting significantly higher cytotoxicity compared to CD19 CAR-NK or CD20 CAR-NK cells. This enhanced efficacy may be attributed to the heightened and more rapid degranulation levels observed in CD19/CD20 dual-targeted CAR-NK cells compared to single-targeted CAR-NK cells. Our study establishes a solid experimental foundation, paving the way for further clinical research to validate the therapeutic potential of dual-target CAR-NK cells in leukemia patients.

### Supplementary Information

**Additional file 1: Figure S1. a** Flow cytometry gating strategy of NK cells. Nucleated cell gates were gated based on sample size and complexity (FCS-A and SSC-A, respectively). Nucleated cells were further gated in FSC-A and FSC-H to screen single cells and exclude double cells. CD3^−^NK cells were gated based on single-cell gates, and CD56^+^ CD16^+^ NK cells were identified using CD56-PE and CD16-APC, and gated based on CD3^−^NK (SSC-A and CD3-FITC). **b** Expression of CD19 and CD20 antigens on different blood tumor cells. **c** Cytotoxicity of UCB-NK cells to different blood tumor cells under different effector-target ratios. (* *p* < 0.05, ** p < 0.01, *** *p* < 0.001, n = 3). **d** Expression of NK cell activity marker CD69 at different times during culture. **Figure S2.** Detection of CAR expression on NK cells after different concentrations of mRNA electroporation (rest for 8 h after electroporation). **Figure S3. a** Levels of perforin, IFN-γ, and IL-15 secreted by NK and CAR-NK cells in the supernatant of 4 h cultures as detected by ELISA (n = 3; * *p* < 0.05, ** *p* < 0.01, *** *p* < 0.001). **b** Expression of CD69 on NK/CAR-NK cells as detected by flow cytometry. **c** Cytokine production of CAR-NK cells as analyzed by flow cytometry. Lymphocyte cells were determined by forward and side scatter and then gated to single cells. They were further gated to CD56^+^ NK cells, and flow cytometry analysis was performed for CD107a, CD69, and IFN-γ in CD56^+^ NK cells.

## Data Availability

Data supporting the findings of this study are included in the published article and its supplementary materials. Additional data are available from the corresponding author upon reasonable request.

## Abbreviations

CAR: Chimeric antigen receptor
NK: Natural killer cells
scFv: Single chain fragment variable
UCB: Umbilical cord blood
ALL: Acute lymphoblastic leukemia
IFN-γ: Interferon-gamma
IL-15: Interleukin 15
R/R-ALL: Refractory and relapse ALL
GVHD: Graft-versus-host disease
CRS: Cytokine release syndrome
ICANS: Immune effector cell-associated neurotoxicity syndrome
MHC: Major histocompatibility complex
TNF-α: Tumor necrosis factor alpha
GM-CSF: Granulocyte–macrophage colony stimulating factor
IVT: in vitro Transcription
ATCC: American tissue culture collection
UCB-MNCs: Umbilical cord blood mononuclear cells
VL: Variable light chain
VH: Variable heavy chain
PI: Propidium Iodide
CFSE: 5,6-Carboxyflu-orescein diacetate, succinimidyl ester
ELISA: Enzyme-linked immunosorbent assay
DAP 10/12: DNAX activation protein 10/12
PB: Peripheral blood
hESCs: Human embryonic stem cells
iPSCs: Induced pluripotent stem cells
HLA: Human leukocyte antigen
K562-MBIL-21: Gene-modified K562 feeder cells expressing membrane-bound IL-21
