# Supplementary material for: CD19/CD20 dual-targeted chimeric antigen receptor-engineered natural killer cells exhibit improved cytotoxicity against acute lymphoblastic leukemia
Source: J Transl Med. 2024 Mar 13;22:274. doi: 10.1186/s12967-024-04990-6 (PMC10935961; doi:10.1186/s12967-024-04990-6)
Supplement: Supplementary file 1 — Additional file 1: Figure S1. a Flow cytometry gating strategy of NK cells. Nucleated cell gates were gated based on sample size and complexity (FCS-A and SSC-A, respectively). Nucleated cells were further gated in FSC-A and FSC-H to screen single cells and exclude double cells. CD3−NK cells were gated based on single-cell gates, and CD56+ CD16+ NK cells were identified using CD56-PE and CD16-APC, and gated based on CD3−NK (SSC-A and CD3-FITC). b Expression of CD19 and CD20 antigens on different blood tumor cells. c Cytotoxicity of UCB-NK cells to different blood tumor cells under different effector-target ratios. (* p < 0.05, ** p < 0.01, *** p < 0.001, n = 3). d Expression of NK cell activity marker CD69 at different times during culture. Figure S2. Detection of CAR expression on NK cells after different concentrations of mRNA electroporation (rest for 8 h after electroporation). Figure S3. a Levels of perforin, IFN-γ, and IL-15 secreted by NK and CAR-NK cells in the supernatant of 4 h cultures as detected by ELISA (n = 3; * p < 0.05, ** p < 0.01, *** p < 0.001). b Expression of CD69 on NK/CAR-NK cells as detected by flow cytometry. c Cytokine production of CAR-NK cells as analyzed by flow cytometry. Lymphocyte cells were determined by forward and side scatter and then gated to single cells. They were further gated to CD56+ NK cells, and flow cytometry analysis was performed for CD107a, CD69, and IFN-γ in CD56+ NK cells. [file 12967_2024_4990_MOESM1_ESM.docx]

**Supplementary information**

**CD19/CD20 dual-targeted chimeric antigen receptor-engineered natural killer cells exhibit improved cytotoxicity**

**against acute lymphoblastic leukemia**

Na Yang[^1^](http://orcid.org/0000-0003-3683-3138)^,2^ [,](http://orcid.org/0000-0003-3683-3138) Caili Zhang^1^, Yingchun Zhang^3^ , Yuting Fan^1,2^ , Jing Zhang^3^, Xiaojin Lin^1,3^ , Ting Guo^4^ , Yangzuo Gu^5^ , Jieheng Wu^2^, Jianmei Gao^6^ , Xing Zhao^1,2,3*^ , Zhixu He^1,2,7,8*^

^1^ Tissue Engineering and Stem Cell Experiment Center, Guizhou Medical University (GMU), Guiyang, Guizhou, China,

^2^ Department of Immunology, College of Basic Medical Sciences, Guizhou Medical University, Guiyang, Guizhou, China,

^3^ Department of Biology, School of Basic Medical Sciences, Guizhou Medical University, Guiyang, Guizhou, China,

^4^ Department of Gynecology, the Affiliated Hospital of Guizhou Medical University, Guiyang, China,

^5^ State Key Laboratory of Biotherapy and Cancer Center, Sichuan University, Chengdu, China,

^6^ School of Pharmacy; Zunyi Medical University, Zunyi, China,

^7^ Key Laboratory of Adult Stem Cell Translational Research (Chinese Academy of Medical Sciences), Guiyang, China,

^8^ Department of Pediatrics, the Affiliated Hospital of Zunyi Medical University, Zunyi, China.

*** CORRESPONDENCE**

Xing Zhao: Email: [xingzhao@gmc.edu.cn](mailto:xingzhao@gmc.edu.cn)

Zhixu He: Email: zxhe@zmu.edu.cn

**
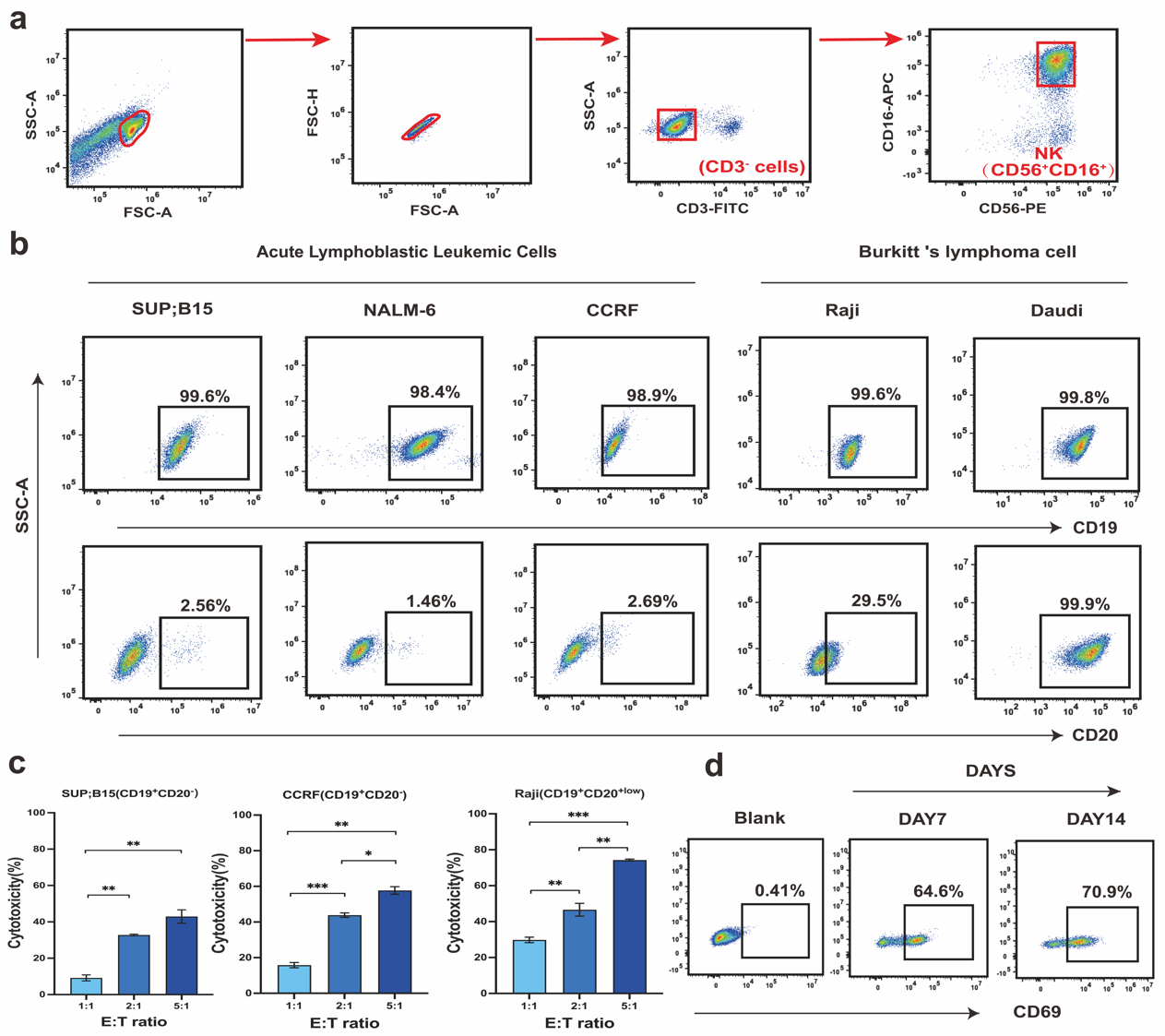
**

**Fig. S1** **a** Flow cytometry gating strategy of NK cells. Nucleated cell gates were gated based on sample size and complexity (FCS-A and SSC-A, respectively). Nucleated cells were further gated in FSC-A and FSC-H to screen single cells and exclude double cells. CD3^-^NK cells were gated based on single-cell gates, and CD56^+^ CD16^+^ NK cells were identified using CD56-PE and CD16-APC, and gated based on CD3^-^NK (SSC-A and CD3-FITC). **b** Expression of CD19 and CD20 antigens on different blood tumor cells. **c** Cytotoxicity of UCB-NK cells to different blood tumor cells under different effector-target ratios. (* *p* < 0.05, ** *p* < 0.01, *** *p* < 0.001, n = 3). **d** Expression of NK cell activity marker CD69 at different times during culture.


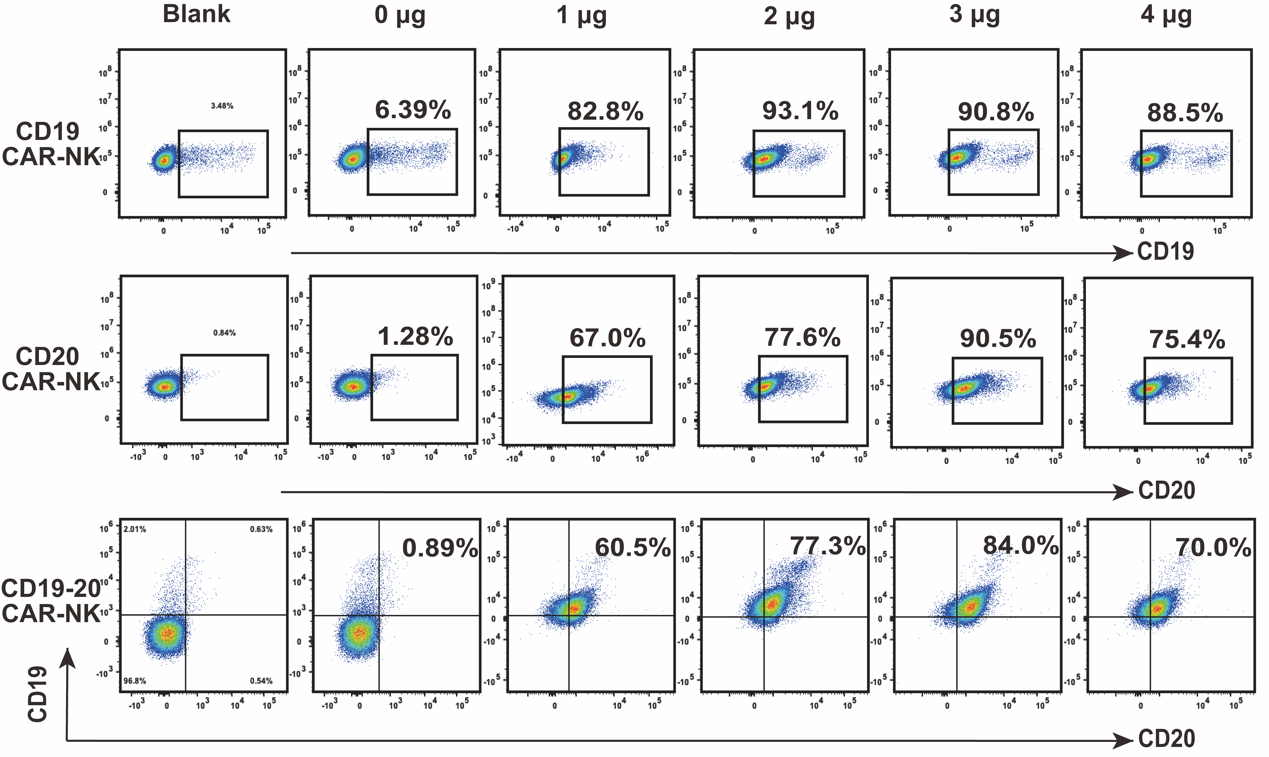


**Fig. S2** Detection of CAR expression on NK cells after different concentrations of mRNA electroporation (rest for 8 hours after electroporation).


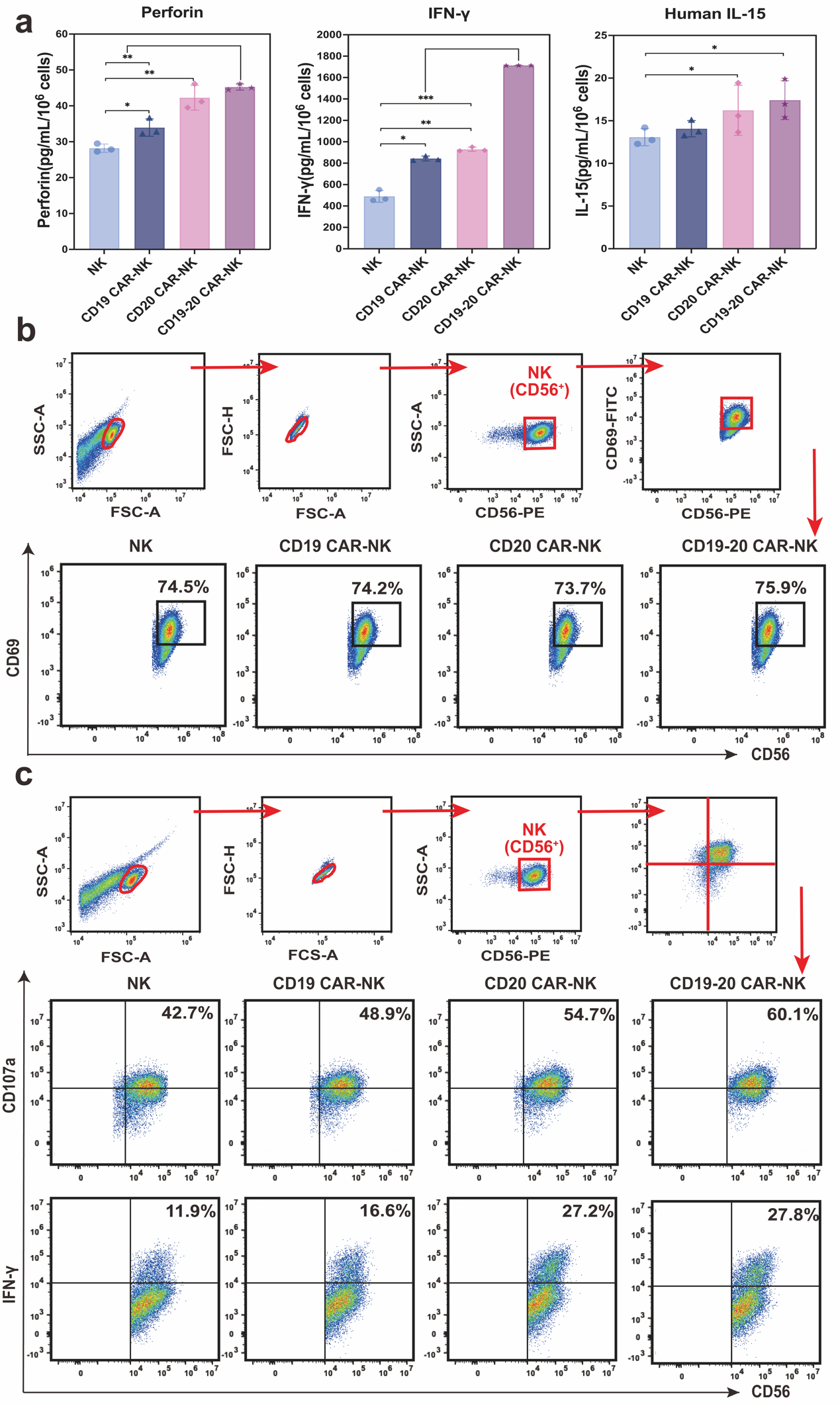


**Fig. S****3** **a** Levels of perforin, IFN-γ, and IL-15 secreted by NK and CAR-NK cells in the supernatant of 4 h cultures as detected by ELISA (n = 3; * *p* < 0.05, ** *p* < 0.01, *** *p* < 0.001). **b** Expression of CD69 on NK/CAR-NK cells as detected by flow cytometry. **c** Cytokine production of CAR-NK cells as analyzed by flow cytometry. Lymphocyte cells were determined by forward and side scatter and then gated to single cells. They were further gated to CD56^+^ NK cells, and flow cytometry analysis was performed for CD107a, CD69, and IFN-γ in CD56^+^ NK cells.
